# Prognostic Factors and Models for Elderly (≥70 Years Old) Primary Operable Triple-Negative Breast Cancer: Analysis From the National Cancer Database

**DOI:** 10.3389/fendo.2022.856268

**Published:** 2022-03-17

**Authors:** Zhuowei Tang, Yuzhu Ji, Yu Min, Xiaohong Zhang, Weiyun Xu, Lijuan Zhao, Jing Zhang, Li Long, Jing Feng, Yixue Wen

**Affiliations:** ^1^ Department of Breast Surgery, Mianyang Central Hospital, School of Medicine, University of Electronic Science and Technology of China, Mianyang, China; ^2^ Department of Pathology, Mianyang Central Hospital, School of Medicine, University of Electronic Science and Technology of China, Mianyang, China; ^3^ Department of Breast and Thyroid Surgery, The Second Affiliated Hospital of Chongqing Medical University, Chongqing, China

**Keywords:** triple-negative breast cancer, overall survival, retrospective study, nomogram, cancer-specific survival

## Abstract

**Background:**

Triple-negative breast cancer (TNBC) is an aggressive subtype of breast cancer. In the elderly (≥70 years old) primary operable (T_1-3_N_0-1_M_0_) TNBC, individualized treatment modalities for this population are pivotal and important, but limited studies are explored.

**Methods:**

The clinicopathological features of elderly primary operable TNBC patients were retrospectively selected from the Surveillance, Epidemiology, and End Results (SEER) database between January 2010 and December 2015. Kaplan–Meier curves were used to show the survival patterns in the different subgroups. Multivariate Cox analysis was used to identify independent risk factors in the 3-, 5-, and 7- year overall survival (OS) and cancer-specific survival (CSS) in this subpopulation. The predictive model was further developed and validated for clinical use.

**Result:**

Between 2010 and 2015 years, a total of 4,761 elderly primary operable TNBC patients were enrolled for the study, with a mean age of 76 years and a median follow-up of 56 months. The multivariate Cox analysis showed that age (increased per year: hazard ratio (HR) = 1.05), race (Asian/Pacific Islander and American Indian/Alaska Native, HR = 0.73), differentiation grade (grade II: HR = 2.01; grade III/IV: HR = 2.67), larger tumor size (T_1c_: HR = 1.83; T_2_: HR = 2.78; T_3_: HR = 4.93), positive N stage (N_1mi_: HR = 1.60; N_1_: HR = 1.54), receiving radiation therapy (HR = 0.66), and receiving adjuvant chemotherapy (HR = 0.61) were the independent prognostic factors for OS, and a similar prognostic pattern was also determined in CSS. Besides, two nomograms for predicting the 3-, 5-, and 7-year OS and CSS in this population were developed with a favorable concordance index of 0.716 and 0.746, respectively.

**Conclusion:**

The results highlight that both radiation and adjuvant chemotherapy are significantly associated with favorable long-term OS and CSS probability in elderly primary operable TNBC patients. Based on the determined independent prognostic factors, the novel nomograms could assist the oncologists to make individualized clinical decisions for the subpopulation at different risks.

## Introduction

Nowadays, breast cancer has become the most frequently diagnosed malignancy and one of the leading causes of cancer-specific death in industrialized countries, with a female predominance (1–4). Nearly 40% of breast cancers occur in patients aged over 65 years and 25% in patients aged over 70 years. As the global population ages, the number of older patients with breast cancer will continue to increase (2, 4). Therefore, breast cancer in the elderly will represent a major public health issue during the next decades. Despite the biological invasive characteristics in older patients being less aggressive than younger breast cancer (5–7), outcomes for older patients with breast cancer are highly variable due to not only several biological factors but also potentially mutable factors (8–10). Thus, there is also a growing number of clinical treatment problems from these patient subgroups including but not limited to young, old, obese, and male breast cancer who often have unique clinical information and who are at high risk for disparate prognostic outcomes (6).

Triple-negative breast cancer (TNBC) accounts for 10%–15% of all breast cancer cases, which lack estrogen and progesterone receptors and express low levels of human epidermal growth factor 2 (Her-2) and therefore do not respond to hormonal or anti-HER2 therapies. Compelling evidence has demonstrated that TNBC frequently implies more aggressive biology and shows a worse prognosis that requires optimal treatment to reduce the future risk of recurrence and mortality (11, 12). For instance, based on the evidence from the large Epidemio-Strategy-Medical-Economical (ESME) metastatic breast cancer cohort, Gobbini et al. reported that there was no improvement in overall survival (OS) of metastatic TNBC patients over the past decades and yielded the need for new strategies in this unique molecular subtype (13). Also, with 390 cases involved, Gal et al. determined that women aged >75 years with TNBC had the highest recurrence rates, the shortest OS probability, and the subsequent worst clinical outcome (14).

Regarding this special subpopulation, however, there are limited data to make appropriate recommendations for those ≥70 years of age. Recently, many well-designed trials and comprehensive reviews demonstrated that adjuvant chemotherapy and radiotherapy are effective at reducing TNBC recurrences and associated with better cancer-specific survival (CSS) and OS in early-stage or younger TNBC patients (11, 15–19). Nevertheless, evidence-based data on the best treatment approach to the elderly patient group are mostly lacking, partly owing to the underrepresentation of elderly patients in clinical studies (9, 20, 21). Moreover, the favorable role of adjuvant chemotherapy in promoting postoperative survival in elderly TNBC patients is still in conflict, with most studies limited to subgroup analyses or small retrospective studies (21–23).

Hereby, the purpose of this study is to explore the impact of radiation therapy or chemotherapy after surgery on the long-term OS and CSS in the setting of elderly primary operable TNBC patients. Besides, we also aim to explore the independent prognostic factors for OS and CSS in this subpopulation and further establish a utility nomogram for oncologists to make tailored clinical decisions.

## Materials and Methods

### Data Source

As an observational retrospective cohort study, patients’ clinical information was extracted from one national cancer database (Surveillance, Epidemiology, and End Results, SEER, derived from the 18 cancer registries), which covered approximately 28% of the US population and is grouped in various races and ethnicities. In 2010, SEER registries began collecting Her-2 receptor status for breast cancer cases (24). Thus, the period of data collection was from 2010 to 2015 years. The reporting of this study has followed the guidelines of the Strengthening the Reporting of Observational Studies in Epidemiology (STROBE) statement (25).

**Figure 1:**
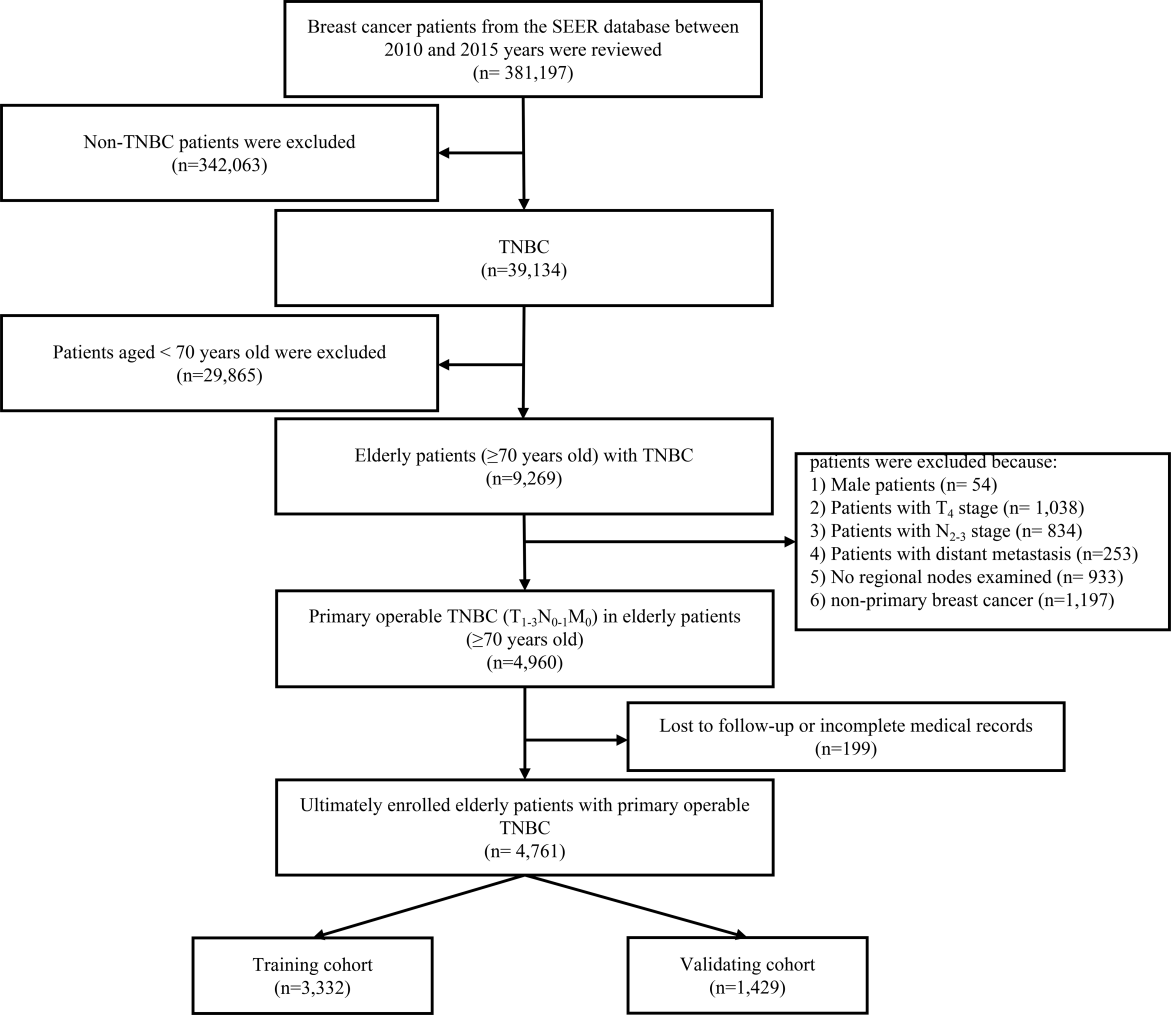
The flow diagram of the patient selection process. TNBC, triple-negative breast cancer.

TNBC was defined by the absence of estrogen receptor α (ERα), progesterone receptor (PR), and Her-2. The age cutoff for breast cancer in the elderly was assigned based on what has been used in previous studies (26–28). Patients who met the following criteria included the following: 1) female patients with postoperative histological confirmed TNBC; 2) age at diagnosis ≥70 years; and 3) the TNM stage classification limited to T_1-3_N_0-1_M_0_. The excluding criteria were as follows: 1) patients with T_4_ (invasion to the chest wall/skin and inflammatory carcinoma) primary site; 2) no regional nodes examined; and 3) lost to follow-up or incomplete medical records. The flow diagram was presented in the study (**Figure 1**).

To perform the multivariate Cox regression analysis, the sample size in this study should be at least 10 times the number of independent variables in the equation. Thus, after excluding the unqualified cases, there were 4,761 elderly female patients with primary operable TNBC enrolled in this study. Moreover, for predicting 3-, 5-, and 7- year OS and CSS probability, the original cohort was randomly divided into a training group and validating cohort at a ratio of 7:3 *via* the “R” program.

### Ethical Approval

Data analysis from this database is considered to be non-human subjects by the Office for Human Research Protection as part of the US Department of Health and Human Services because patient data were anonymized and publicly available. For these reasons, the need for ethics approval was omitted by the Mianyang Central Hospital Ethics Committee.

### Variable Evaluation and Definition

Variables were extracted based on their associations with the prognosis outcomes of interest. Namely, the following clinicopathological features were collected and transformed into categorical variables: race (White, Black, other including Asian or Pacific Islander, and American Indian/Alaska Native), laterality (right and left origin of primary), stage (IA, IB, IIA, IIB, and IIIA deriving from the adjusted AJCC staging system 7th edition), grade (I: well differentiated, II: moderately differentiated, III/IV: poorly differentiated and undifferentiated), tumor location (nipple, central, outer, inner, overlapping and axillary of breast), histological subtype (IDC, ILC, and other kinds of subtypes), primary tumor stage (T_1mi_: >0 and ≤1 mm, T_1a_: >1 and ≤5 mm, T_1b_: >5 and ≤10 mm, T_1c_: >10 and ≤20 mm, T_2_: >20 and ≤50 mm; T_3_: >50 mm), lymph node stage (N_0_: no regional lymph node metastasis identified or isolated tumor cell; N_1micro_: micrometastases: approximately 200 cells, larger than 0.2 mm, but none larger than 2.0 mm; N_1macro_: metastasis in 1–3 axillary lymph nodes, and/or in clinical negative internal mammary nodes with micrometastases or macrometastases by sentinel lymph node biopsy), primary surgical extension (partial/less than total mastectomy: includes segmental mastectomy, lumpectomy, quadrantectomy, tylectomy, wedge resection, nipple resection, excisional biopsy, or partial mastectomy; modified radical/total mastectomy), postoperative radiation record (performed or not), and chemotherapy recode (performed or not). The OS and CSS probability were calculated in months (more than 0 days of survival). The age at diagnosis was used as a continuous variable.

### Statistical Analysis

The primary endpoint of this observational retrospective study was the 3-, 5-, and 7-year OS and CSS. The secondary endpoint was the efficacy of radiation and chemotherapy in the prognosis of the elderly primary operable TNBC patients. The univariate and multivariate Cox regression analyses were performed to find out the independent prognostic factors of OS and CSS in elderly primary operable TNBC patients. A two-tailed p-value of <0.05 was considered significant. Age, chemotherapy, radiation therapy, and factors significant in the univariate analysis are defined as the criterion for performing backward stepwise selection. The nomogram, decision curve analysis (DCA), calibration curve, and Kaplan–Meier analysis were constructed and plotted based on the results (availability, importance, and clinical relevance) derived from the multivariate Cox regression analysis *via* using the “survival,” “rms,” “survminer,” and “foreign” packages of the R software (R Foundation, Vienna, Austria, version 4.0.3, http://www.r-project.org). Harrell’s C-index (29) and the time-dependent area under the receiver (AUC) operating characteristic (ROC) curve are conducted to assess the discrimination performance of the present nomogram.

## Results

### Clinicopathological Characteristics of Elderly Primary Operable TNBC Patients

In total, from the SEER database between 2010 and 2015 years, 4,761 elderly primary operable TNBC patients were enrolled in this study with a mean age of 76.85 years at diagnosis and a median follow-up time of 56 months (range: 1–107 months). White race played a majority population in the present study (3,742 cases, 78.6%), whereas Asian or Pacific Islander and American Indian/Alaska Native only accounted for 3.4% of the whole population (340 cases). Based on the TNM stage classification, nearly half of the study population in the present study was at the IA stage (2,365 cases, 49.7%). The patients were subsequently randomized divided into training (3,332 cases) and validating (1,429 cases) cohorts for further Cox analysis and nomogram construction as well validation. The specific demographic and clinical characteristics of the elderly primary operable TNBC patients are shown in **Table 1**.

**Table 1:** Clinicopathological characteristics of elderly primary operable TNBC patients (≥70 years old) in training and validation cohorts.

Characteristics	No. (%) of patients		
	Initial cohort (n = 4,761)	Training cohort (n = 3,332)	Validation cohort (n = 1,429)
**Age**	76.85 ± 5.54*^c^*	76.87 ± 5.64	76.81 ± 5.49
**Race**			
White	3,742 (78.6)	2,642 (79.3)	1,100 (77.0)
Black	705 (14.8)	471 (14.1)	234 (16.4)
Other*^a^*	314 (6.6)	219 (6.6)	95 (6.6)
**Location**			
Nipple	14 (0.3)	8 (0.2)	6 (0.4)
Central	218 (4.6)	156 (4.7)	62 (4.3)
Upper-inner	690 (14.5)	489 (14.7)	201 (14.1)
Lower-inner	326 (6.8)	216 (6.5)	110 (7.7)
Upper-outer	1,888 (39.7)	1,308 (39.2)	580 (40.6)
Lower-outer	398 (8.3)	289 (8.8)	109 (7.6)
Axillary	28 (0.6)	22 (0.6)	6 (0.4)
Overlapping	1,199 (25.2)	844 (25.3)	355 (24.8)
**^&^Grade**			
I	166 (3.5)	120 (3.6)	46 (3.2)
II	1,201 (25.2)	823 (24.7)	378 (26.5)
III/IV	3,394 (71.3)	2,389 (71.7)	1,005 (70.3)
**Laterality**			
Right	2,307 (48.5)	1,603 (48.1)	704 (49.3)
Left	2,454 (51.5)	1,729 (51.9)	725 (50.7)
**Histology**			
IDC	4,169 (87.6)	2,912 (87.4)	1,257 (88.0)
ILC	74 (1.5)	48 (1.4)	26 (1.8)
^#^Other	518 (10.9)	372 (11.2)	146 (10.2)
**T stage**			
T_mi+1a_	288 (6.0)	208 (6.2)	80 (5.6)
T_1b_	728 (15.3)	505 (15.2)	223 (15.6)
T_1c_	1,689 (35.5)	1,176 (35.3)	513 (35.9)
T_2_	1,811 (38.0)	1,265 (38.0)	546 (38.2)
T_3_	245 (5.2)	178 (5.3)	67 (4.7)
**N stage**			
N_0_	3,692 (77.5)	2,568 (77.1)	1,124 (78.7)
N1_mi_	287 (6.1)	202 (6.1)	85 (5.9)
N_1_	782 (16.4)	562 (16.8)	220 (15.4)
**AJCC 7th stage**			
IA	2,365 (49.7)	1,638 (49.2)	727 (50.9)
IB	77 (1.6)	60 (1.8)	17 (1.2)
IIA	1,531 (32.2)	1,071 (32.1)	460 (32.2)
IIB	687 (14.4)	487 (14.6)	200 (14.0)
IIIA	101 (2.1)	76 (2.3)	25 (1.7)
**Surgical extension**			
Less than total mastectomy	2,843 (59.7)	1,994 (60.0)	849 (59.4)
Modified radical/total mastectomy	1,918 (40.3)	1,338 (40.0)	580 (40.6)
**Radiation**			
Not performed	2,256 (47.4)	1,565 (47.0)	691 (48.4)
Performed	2,505 (52.6)	1,767 (53.0)	738 (51.6)
**Chemotherapy**			
Not performed	2,686 (56.4)	1,858 (55.8)	828 (57.9)
Performed	2,075 (43.6)	1,474 (44.2)	601 (42.1)
**RLN harvested**	4.95 ± 5.26*^c^*	4.91 ± 5.24	4.97 ± 5.40
**RLN positive**	0.33 ± 0.94*^c^*	0.32 ± 0.88	0.33 ± 1.05
**Marital status**			
Married	2,069 (43.5)	1,446 (43.4)	623 (43.6)
Divorce	456 (9.5)	310 (9.3)	146 (10.2)
Single	2,012 (42.3)	1,421 (42.6)	591 (41.4)
Unknown	224 (4.7)	155 (4.6)	69 (4.8)
**Postoperative follow-up**	56 [1–107]^¶^	56 [1–107]^¶^	56 [1–107]^¶^

^a^other: defined as the Asian/Pacific Islander and American Indian/Alaska Native.

^&^Grade: I: well differentiated, II: moderately differentiated, III/IV: poorly differentiated and undifferentiated.

^c^Mean ± SD.

^¶^Median [range].

TNBC, triple-negative breast cancer; IDC, invasive ductal carcinoma; ILC, invasive lobular carcinoma; ^#^other, other types of breast cancer; RLN, regional lymph node.

### Kaplan–Meier Curves of Radiotherapy and Chemotherapy in OS and CSS

**Figure 2:**
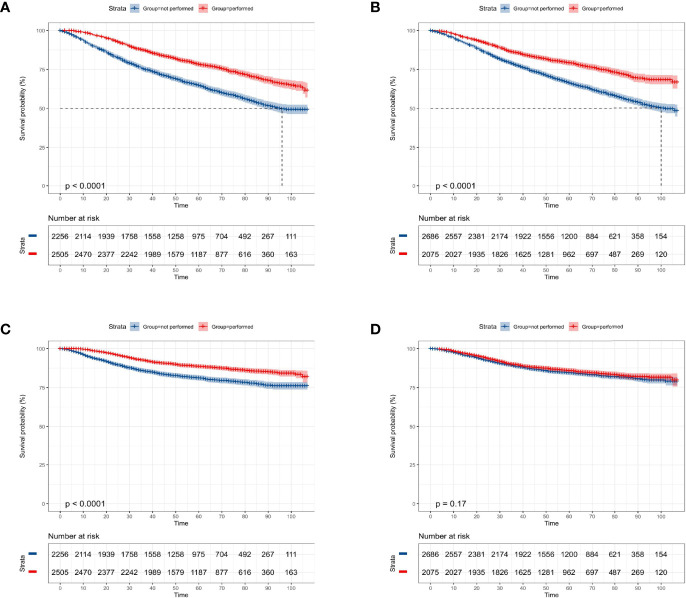
Kaplan–Meier overall survival and cancer-specific survival analyses of 4,761 women aged 70 years or older with primary operable, triple-negative breast cancer regarding the adjuvant treatment. **(A)** Radiotherapy for OS; **(B)** chemotherapy for OS; **(C)** radiotherapy for CSS; **(D)** chemotherapy for CSS. OS, overall survival; CSS, cancer-specific survival. Tick marks indicate censored data.

**Figure 3:**
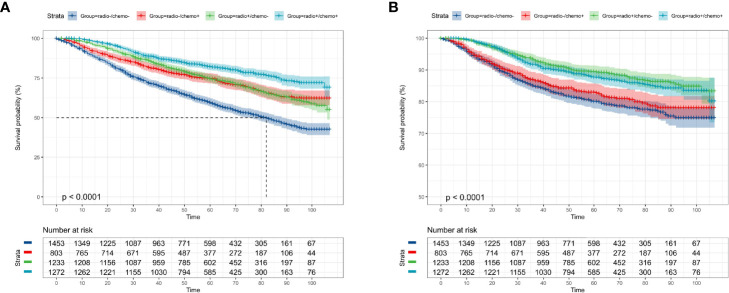
Kaplan–Meier overall survival and cancer-specific survival analyses of 4,761 women aged 70 years or older with primary operable, triple-negative breast cancer who received both chemotherapy and radiotherapy (radio+/chemo+), received only radiotherapy (radio+/chemo-), received only chemotherapy (radio-/chemo+), or did not receive radiotherapy and chemotherapy (radio-/chemo-). **(A)** adjuvant treatment for OS; **(B)** adjuvant treatment for CSS. OS, overall survival; CSS, cancer-specific survival. Tick marks indicate censored data.

Among the whole study population, approximately 47.4% (2,256 cases) of patients were not elected to receive radiation therapy and a similar result was found in the chemotherapy record (2,686 cases, 56.4%). The KM curves presented that receiving radiation therapy could benefit the OS (p < 0.0001, **Figure 2A**) and CSS probability (p < 0.0001, **Figure 2C**) of the elderly primary operable TNBC patients during the follow-up, compared with patients not assigned to radiation therapy. On the other hand, patients who received chemotherapy had significantly higher 3-, 5-, and 7-year OS probability (p < 0.0001, **Figure 2B**), while it did not show any statistically significant CSS benefit (p = 0.17, **Figure 2D**). In the subgroup analysis, patients who did not receive any chemoradiotherapy had the worst survival outcome (OS: p < 0.0001, **Figure 3A**; CSS: p < 0.0001, **Figure 3B**).

### Kaplan–Meier Curves of Clinicopathological Characteristics in OS and CSS

**Figure 4:**
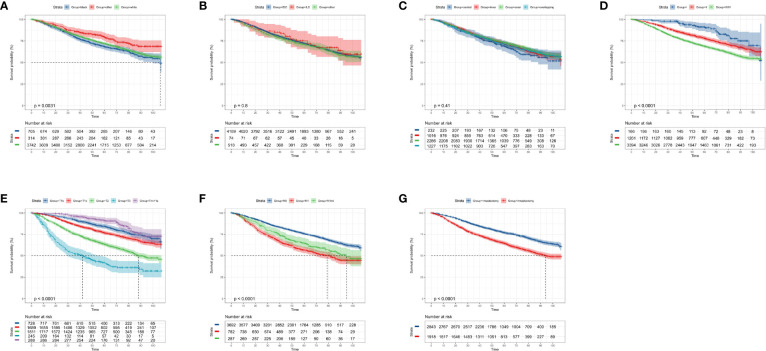
Kaplan–Meier overall survival analysis of 4,761 women aged 70 years or older with primary operable, triple-negative breast cancer regarding the clinicopathological characteristics. **(A)** Race. **(B)** Histology. **(C)** Tumor location. **(D)** Differentiation grade. **(E)** T stage. **(F)** N stage. **(G)** Surgical extension. other: defined as the Asian/Pacific Islander and American Indian/Alaska Native; Grade: I: well-differentiated, II: moderately differentiated, III/IV: poorly differentiated and undifferentiated; central: central portion of breast combined with nipple; other: other types of breast cancer. TNBC, triple-negative breast cancer; IDC, invasive ductal carcinoma; ILC, invasive lobular carcinoma. Tick marks indicate censored data.

**Figure 5:**
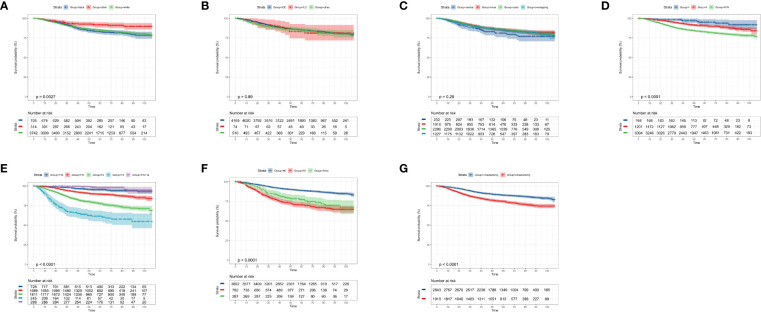
Kaplan–Meier cancer-specific survival analysis of 4,761 women aged 70 years or older with primary operable, triple-negative breast cancer regarding the clinicopathological characteristics. **(A)** Race. **(B)** Histology. **(C)** Tumor location. **(D)** Differentiation grade. **(E)** T stage. **(F)** N stage. **(G)** surgical extension. other: defined as the Asian/Pacific Islander and American Indian/Alaska Native; Grade: I: well-differentiated, II: moderately differentiated, III/IV: poorly differentiated and undifferentiated; central: central portion of breast combined with nipple; other: other types of breast cancer. TNBC, triple-negative breast cancer; IDC, invasive ductal carcinoma; ILC, invasive lobular carcinoma. Tick marks indicate censored data.

According to the KM curves, a significant decrease in cumulative OS probability was observed in patients with black or white race (p = 0.0031, **Figure 4A**), worse differentiation grade (III/IV, p < 0.0001, **Figure 4D**), larger primary tumor size (T_1c_, T_2_, and T_3_; p < 0.0001, **Figure 4E**), late N stages (N_1mi_ and N_1ma_; p < 0.0001, **Figure 4F**), and relatively aggressive surgical extension (modified radical/total mastectomy, p < 0.0001, **Figure 4G**). On the contrary, tumor subtype (p = 0.800, **Figure 4B**) and tumor location (p = 0.410, **Figure 4C**) were not associated with the OS in elderly primary operable TNBC patients. Regarding the CSS, a similar survival pattern was observed in the KM curves (**Figures 5A–G**).

### Univariate and Multivariate Cox Analyses of the Prognostic Factors for OS

In terms of 3-, 5-, and 7- year OS, univariate Cox analysis showed that age (increased per year: hazard ratio (HR) = 1.08, 95% confident interval (CI): 1.07–1.09; p ≤ 0.001), worse differentiation grade (grade II: HR = 2.31, 95% CI: 1.34–3.97; grade III/IV: HR = 3.37, 95% CI: 1.98–5.72, p < 0.001), larger tumor size (T_1c_: HR = 1.87, 95% CI: 1.30–2.70; T_2_: HR = 3.30, 95% CI: 2.31–4.73, T_3_: HR = 6.81, 95% CI: 4.58–10.12, p < 0.001), positive N stage (N_1mi_: HR = 1.65, 95% CI: 1.31–2.07; N_1_: HR = 1.91, 95% CI: 1.65–2.21, p < 0.001), and radical surgical extension (HR = 1.76, 95% CI: 1.55–1.98) were the potential risk factors in impairing the long-term OS probability. On the contrary, Asian/Pacific Islander and American Indian/Alaska Native race (HR = 0.76, 95% CI: 0.58–1.01, p = 0.025), receiving radiation therapy (HR = 0.51, 95% CI: 0.45–0.58, p < 0.001), and receiving chemotherapy (HR = 0.56, 95% CI: 0.49–0.64, p < 0.001) were favorable prognostic factors for OS (**Table 2**).

**Table 2:** Univariate and multivariate Cox regression analyses of predictive variables correlated with OS in elderly primary operable TNBC patients (≥70 years old).

Variables	Subgroup	Univariable		Multivariable	
		Hazard ratio	*p*	Hazard ratio	*p*
**Age (year)**	Per year	1.08 (1.07–1.09)	<0.001	1.05 (1.04–1.06)	<0.001
**Race**	White	Reference	0.025	Reference	0.029
	Black	1.16 (0.98–1.38)		1.19 (0.93–1.31)	
	Other*^a^*	0.76 (0.58–1.01)		0.73 (0.55–0.96)	
**Location**	Central*^b^*	Reference	0.657	/
	Inner	0.84 (0.63–1.13)		
	Outer	0.84 (0.64–1.10)		
	Overlap	0.87 (0.65–1.15)		
**Grade**	I	Reference	<0.001	Reference	<0.001
	II	2.31 (1.34–3.97)		2.01 (1.16–3.45)	
	III/IV	3.37 (1.98–5.72)		2.67 (1.57–4.55)	
**Histology**	IDC	Reference	0.836	/
	ILC	0.94 (0.56–1.56)		
	Other	1.05 (0.87–1.27)		
**T stage**	T_mi+1a_	Reference	<0.001	Reference	<0.001
	T_1b_	1.29 (0.86–1.92)		1.28 (0.85–1.91)	
	T_1c_	1.87 (1.30–2.70)		1.83 (1.26–2.64)	
	T_2_	3.30 (2.31–4.73)		2.78 (1.93–4.04)	
	T_3_	6.81 (4.58–10.12)		4.93 (3.26–7.48)	
**N stage**	N_0_	Reference	<0.001	Reference	
	N_1mi_	1.65 (1.31–2.07)		1.60 (1.27–2.01)	<0.001
	N_1_	1.91 (1.65–2.21)		1.54 (1.32–1.79)	
**Surgical extension**	<Mastectomy	Reference	<0.001	Reference	0.406
	≥Mastectomy	1.76 (1.55–1.98)		0.93 (0.80–1.09)	
**Radiation**	Not	Reference	<0.001	Reference	<0.001
	performed	0.51 (0.45–0.58)		0.66 (0.56–0.77)	
**Chemotherapy**	Not	Reference	<0.001	Reference	<0.001
	performed	0.56 (0.49–0.64)		0.61 (0.52–0.71)	

a Other: defined as the Asian/Pacific Islander and American Indian/Alaska Native.

b Grade: I: well differentiated, II: moderately differentiated, III/IV: poorly differentiated and undifferentiated; central: central portion of breast combined with nipple; other: other types of breast cancer.

TNBC, triple-negative breast cancer; IDC, invasive ductal carcinoma; ILC, invasive lobular carcinoma.

Bold values indicate statistical significance (p < 0.05).

In stepwise multivariate Cox analysis, seven factors including age (increased per year: HR = 1.05, 95% CI: 1.04–1.06, p < 0.001), Asian/Pacific Islander and American Indian/Alaska Native race (HR = 0.73, 95% CI: 0.55–0.96, p = 0.029), differentiation grade (grade II: HR = 2.01, 95% CI: 1.16–3.45; grade III/IV: HR = 2.67, 95% CI: 1.57–4.55, p < 0.001), larger tumor size (T_1c_: HR = 1.83, 95% CI: 1.26–2.64; T_2_: HR = 2.78, 95% CI: 1.93–4.02, T_3_: HR = 4.94, 95% CI: 3.27–7.46, p < 0.001), positive N stage (N_1mi_: HR = 1.60, 95% CI: 1.27–2.01; N_1_: HR = 1.54, 95% CI: 1.32–1.79, p < 0.001), receiving radiation therapy (HR = 0.66, 95% CI: 0.56–0.77, p < 0.001), and receiving chemotherapy (HR = 0.61, 95% CI: 0.52–0.71, p < 0.001) were the independent prognostic factors for OS (**Table 2**).

### Univariate and Multivariate Cox Analyses of the Prognostic Factors for CSS

In terms of 3-, 5-, and 7- year CSS, univariate Cox analysis showed that age (increased per year: HR = 1.06, 95% CI: 1.04–1.08; p ≤ 0.001), worse differentiation grade (grade II: HR = 2.50, 95% CI: 1.01–6.16; grade III/IV: HR = 4.61, 95% CI: 1.91–11.14, p < 0.001), larger tumor size (T_1c_: HR = 3.39, 95% CI:.58–7.26; T_2_: HR = 7.89, 95% CI: 3.72–16.71, T_3_: HR = 17.24, 95% CI: 7.89–37.69, p < 0.001), positive N stage (N_1mi_: HR = 2.25, 95% CI: 1.65–3.07; N_1_: HR = 2.86, 95% CI: 2.35–3.48, p < 0.001), and radical surgical extension (HR = 1.90, 95% CI: 1.59–2.27) were the potential risk factor in impairing the long-term CSS. By contrast, Asian/Pacific Islander and American Indian/Alaska Native race (HR = 0.48, 95% CI: 0.29–0.79, p = 0.004) and receiving radiation therapy (HR = 0.51, 95% CI: 0.43–0.62, p < 0.001) were favorable prognostic factors for CSS (**Table 3**). As for chemotherapy, there was a slight trend to become statistically significant (HR = 0.87, 95% CI: 0.73–1.05, p = 0.161).

**Table 3:** Univariate and multivariate Cox regression analyses of predictive variables correlated with CSS in elderly primary operable TNBC patients (≥70 years old).

Variables	Subgroup	Univariable		Multivariable	
		Hazard ratio	*p*	Hazard ratio	*p*
Age (year)	Per year	1.06 (1.04–1.08)	<0.001	1.03 (1.01–1.05)	<0.001
Race	White	Reference	0.004	Reference	0.004
	Black	1.19 (0.93–1.51)		1.08 (0.84–1.37)	
	Other*^a^*	0.48 (0.29–0.79)		0.44 (0.26–0.73)	
Location	Central*^b^*	Reference	0.509	/	
	Inner	0.78 (0.52–1.17)			
	Outer	0.74 (0.50–1.09)			
	Overlap	0.76 (0.51–1.14)			
Grade	I	Reference	<0.001	Reference	0.001
	II	2.50 (1.01–6.16)		1.93 (0.78–4.78)	
	III/IV	4.61 (1.91–11.14)		2.83 (1.17–6.88)	
Histology	IDC	Reference	0.484	/	
	ILC	1.25 (0.65–2.43)			
	Other*^c^*	1.15 (0.87–1.51)			
T stage	T_mi+1a_	Reference	<0.001	Reference	<0.001
	T_1b_	1.51 (0.65–3.50)		1.43 (0.61–3.31)	
	T_1c_	3.39 (1.58–7.26)		2.95 (1.37–6.34)	
	T_2_	7.89 (3.72–16.71)		5.61 (2.62–12.01)	
	T_3_	17.24 (7.89–37.69)		10.61 (4.86–24.09)	
N stage	N_0_	Reference	<0.001	Reference	
	N_1mi_	2.25 (1.65–3.07)		2.03 (1.49–2.78)	<0.001
	N_1_	2.86 (2.35–3.48)		2.06 (1.68–2.53)	
Surgical extension	<Mastectomy	Reference	<0.001	Reference	0.542
	≥Mastectomy	1.90 (1.59–2.27)		0.93 (0.74–1.16)	
Radiation	Not	Reference	<0.001	Reference	<0.001
	performed	0.51 (0.43–0.62)		0.63 (0.50–0.79)	
Chemotherapy	Not	Reference	0.161	Reference	0.035
	performed	0.87 (0.73–1.05)		0.79 (0.64–0.98)	
					

^a^Other: defined as the Asian/Pacific Islander and American Indian/Alaska Native.

^b^Grade: I: well differentiated, II: moderately differentiated, III/IV: poorly differentiated and undifferentiated; central: central portion of breast combined with nipple.

^c^Other: other types of breast cancer.

TNBC ,triple-negative breast cancer; IDC, invasive ductal carcinoma; ILC, invasive lobular carcinoma.

Bold values indicate statistical significance (p < 0.05).

In stepwise multivariate Cox analysis, seven variables including age (increased per year: HR = 1.03, 95% CI: 1.01–1.05, p < 0.001), Asian/Pacific Islander and American Indian/Alaska Native race (HR = 0.44, 95% CI: 0.26–0.73, p = 0.004), differentiation grade (grade III/IV: HR = 2.86, 95% CI: 1.17–6.88, p = 0.001), larger tumor size (T_1c_: HR = 2.95, 95% CI: 1.37–6.34; T_2_: HR = 5.61, 95% CI: 2.62–12.01, T_3_: HR = 10.61, 95% CI: 4.86–24.09, p < 0.001), positive N stage (N_1mi_: HR = 2.03, 95% CI: 1.49–2.87; N_1_: HR = 2.06, 95% CI: 1.68–2.53, p < 0.001), receiving chemotherapy (HR = 0.79, 95% CI: 0.64–0.98, p = 0.035), and radiation therapy (HR = 0.63, 95% CI: 0.50–0.79, p < 0.001) were the independent prognostic factors for CSS (**Table 3**).

### Development and Validation of Nomograms for Predicting the OS and CSS

**Figure 6:**
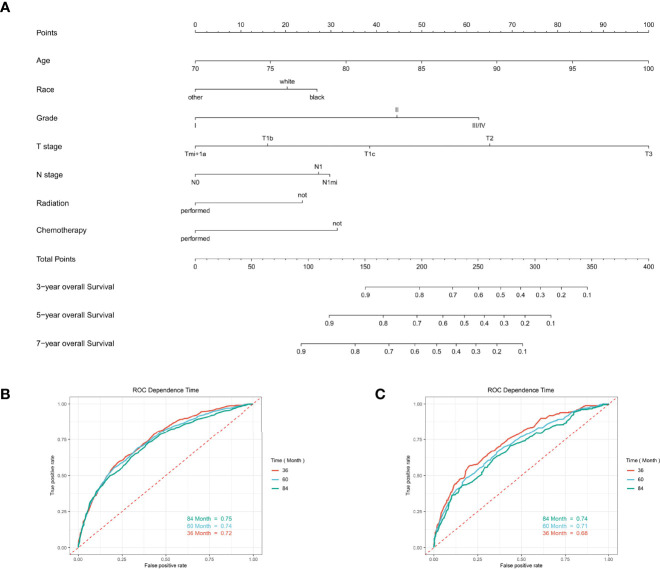
The predictive model for predicting the long-term overall survival probability in women aged 70 years or older with primary operable, triple-negative breast cancer in the training cohort. **(A)** Nomogram for predicting the 3-, 5-, and 7- year OS for elderly primary operable TNBC patients. **(B)** The receiver operating characteristics (ROC) curve and area under the ROC curve (AUC) in the training cohort. **(C)** The receiver operating characteristics (ROC) curve and area under the ROC curve (AUC) validating cohort.

Based on the multivariate Cox regression analysis above, any variable with a significant correlation was included in developing clinical nomogram models (**Figure 6A**). Each factor was given a score on the point scale and the total point could be calculated by adding up all the specific values from an individualized patient (**Tables S1**, **S2**).

**Figure 7:**
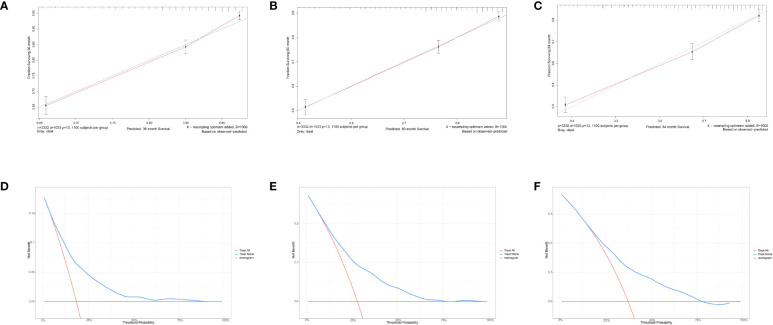
Calibration curves and decision curve analysis for evaluating the accuracy of the nomogram in predicting the overall survival. The solid red line represented the performance of the nomogram, of which the closer fit to the gray line represents the better prediction of the nomogram we constructed. **(A)** 3-year OS in elderly primary operable TNBC patients, **(B)** 5-year OS in elderly primary operable TNBC patients; **(C)** 7-year OS in elderly primary operable TNBC patients; **(D)** DCA for 3-year OS in elderly primary operable TNBC patients in the training cohort; **(E)** DCA for 5-year OS in elderly primary operable TNBC patients in the training cohort; **(F)** DCA for 7-year OS in elderly primary operable TNBC patients in the training cohort. OS, overall survival; DCA, decision curve analysis; TNBC, triple-negative breast cancer.

For predicting the 3-, 5-, and 7- year OS probability, the C-index of the nomogram was 0.716 (95% CI: 0.687–0.751), and the AUC of the 3-, 5-, and 7- year time-dependent ROC reached 0.720, 0.740, and 0.750, respectively (**Figure 6B**). Moreover, to validate the accuracy of our nomogram, an internal validation cohort with 1,429 cases was adopted. The results in the validating cohort presented good discrimination with an AUC of 0.680 in predicting 3-year OS, 0.710 in predicting 5-year OS, and 0.740 in predicting 7-year OS (**Figure 6C**). To evaluate the utility of the nomogram, three calibration curves of the nomogram were displayed. The curves (apparent, ideal, and bias-corrected lines) indicated a high agreement in predicting the 3-, 5-, and 7-year OS (**Figures 7A–C**). The decision curve analysis (DCA) curves presented that the score derived from the nomogram would be more effective than a treat-none or treat-all strategy when the threshold probability reached 75% in three cohorts (**Figures 7D–F**).

**Figure 8:**
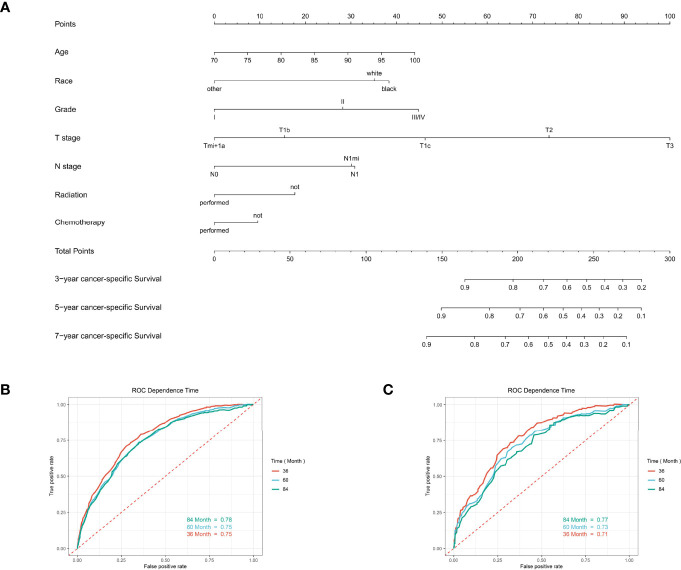
The predictive model for predicting the long-term cancer-specific survival probability in women aged 70 years or older with primary operable, triple-negative breast cancer in the training cohort. **(A)** Nomogram for predicting the 3-, 5-, and 7- year CSS for elderly primary operable TNBC patients. **(B)** The receiver operating characteristics (ROC) curve and area under the ROC curve (AUC) in the training cohort. **(C)** The receiver operating characteristics (ROC) curve and area under the ROC curve (AUC) validating cohort.

**Figure 9:**
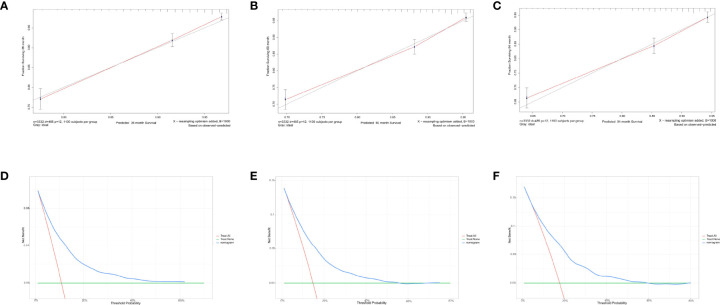
Calibration curves and decision curve analysis for evaluating the accuracy of the nomogram in predicting cancer-specific survival. The solid red line represents the performance of the nomogram, of which the closer fit to the gray line represents the better prediction of the nomogram we constructed. **(A)** 3-year CSS in elderly primary operable TNBC patients; **(B)** 5-year CSS in elderly primary operable TNBC patients; **(C)** 7-year CSS in elderly primary operable TNBC patients; **(D)** DCA for 3-year CSS in elderly primary operable TNBC patients in the training cohort; **(E)** DCA for 5-year CSS in elderly primary operable TNBC patients in the training cohort; **(F)** DCA for 7-year CSS in elderly primary operable TNBC patients in the training cohort. CSS, cancer-specific survival; DCA, decision curve analysis; TNBC, triple-negative breast cancer.

For predicting the 3-, 5-, and 7-year CSS probability, a novel nomogram (**Figure 8A**) was established with a C-index of 0.746 (95% CI: 0.713–0.803) and the AUC of the 3-, 5-, and 7-year time-dependent ROC reached 0.750, 0.750, and 0.780, respectively (**Figure 8B**). Moreover, the AUC of the 3-, 5-, and 7-year time-dependent ROC in the validating cohort was 0.710, 0.730, and 0.770, respectively (**Figure 8C**). Similarly, the calibration curves (**Figures 9A–C**) and DCA (**Figures 9D–F**) suggested the feasibility of the nomogram in applying for clinical use.

## Discussion

With the aging process, an increasing number of older women would be diagnosed with breast cancer and many are diagnosed at stages requiring more aggressive treatment, which needs efforts to increase rates of earlier stage diagnosis and the development of less toxic treatments that could help improve postoperative survival while preserving the quality of life (6, 21, 26, 28, 30). Currently, many clinical trials have demonstrated that elderly women with TNBC had the worst outcome when compared with other subtypes of breast cancer. Regarding the unique molecular subtype of TNBC patients, adjuvant chemotherapy modality, therefore, plays a crucially important role in deescalating the tumor progression and reducing the risk of recurrence as well as cancer-specific death. Unfortunately, the value of adjuvant chemotherapy in old patients with early breast cancer remains controversial (14, 19, 26, 30–32). There is a paucity of data about the benefits of chemotherapy in elderly women with breast cancer (11). Few prospective data exist for chemotherapy in older breast cancer patients (≥70 years old) concerning efficacy or toxicity, but previous studies did suggest that the whole TNBC population could benefit from the adjuvant chemotherapy treatment (26, 33). As for elderly primary operable TNBC patients, whether active or omitting adjuvant treatment could further improve survival rates after local therapy still lacks robust evidence.

Among 4,761 elderly primary operable TNBC patients in our cohort, only 52.6% of them received radiation therapy and less than half of the patients received chemotherapy (43.6%). Notably, the KM curves showed that OS but not CSS benefits from the addition of adjuvant chemotherapy to surgery. Based on literature review (11, 34) and clinical experience, chemotherapy appears to influence the prognosis of TNBC patients. We hereby add this factor for further multivariate analysis, regardless of the p-value derived from the univariate analysis. Remarkably, after adjusting other confounders, the results demonstrated that patients who received chemotherapy presented longer OS and CSS probability. Similarly, in an earlier study derived from the SEER database (30), Elkin et al. determined that adjuvant chemotherapy was associated with a significant reduction in mortality among older women with negative hormone receptor status but lymph node-positive breast cancer. However, some confounders, like Her-2 status, were unavailable at that time. In the present study, we reanalyzed the cases from the latest version of the SEER database (between 2010 and 2015 years) with the target population. We addressed this limitation and further validated and highlighted the beneficial role of adjuvant treatment in reducing the long-term mortality of elderly primary operable TNBC. Most recently, Morita et al. conducted a retrospective multicenter study in Japan (27). However, they did not find any significant difference in OS among older patients who received adjuvant chemotherapy or not (p = 0.333). Alternatively, patients who received adjuvant chemotherapy had significantly prolonged disease-free survival (p = 0.037). The different results of our study and theirs might be contributed to the varied study population (operable TNBC vs. whole breast cancer population) and chemotherapy rate (43.6% vs. 14%).

On the other hand, based on existing evidence, the value of adjuvant radiotherapy in elderly TNBC patients also remains in conflict. One earlier meta-analysis (twelve studies were included within 5,507 TNBC cases) showed that adjuvant radiotherapy was not likely to benefit the OS of the elderly population but women with late-stage disease and younger patients (15). Moreover, in another Asian multicenter comparative study, Bhoo-Pathy et al. also showed that adjuvant radiotherapy was only associated with better survival in locally advanced or very young TNBC patients (16). However, in one study from the SEER database (median follow-up was 45 months), Zhai et al. determined that adjuvant radiotherapy after breast-conserving surgery (BCS) was associated with better OS and CSS in patients aged ≥70 years (35). Collectively, most published studies on the role of adjuvant radiotherapy in improving the OS for elderly patients are either retrospective observational or comparative studies (16, 18, 35). In our study, the effect of radiotherapy was significant for OS as well as CSS of operable TNBC patients, which could reduce the risk of mortality by about half (OS: HR = 0.66; CSS: HR = 0.63). The subgroup analysis indicated that patients receiving both radiotherapy and chemotherapy showed the highest survival probability, whereas patients omitting chemoradiotherapy had the worst OS and CSS. Therefore, prospective randomized controlled studies focused on adjuvant treatment in older breast cancer should be carried out in the future to improve the care quality for this population and the level of evidence-based medicine (11, 17).

In addition, some clinicopathological parameters including tumor differentiation grade, tumor size, and regional lymph node status which were well known associated with the prognosis of TNBC survival were again confirmed in the present study. Interestingly, the primary surgical extension was observed to be a significant predictor for OS and CSS during the univariate analysis. By contrast, the significance of this relationship with survival probability was eliminated in the stepwise multivariate analysis. Besides, while the lymph node stage was a pivotal indicator for the prognosis of elderly primary operable TNBC patients, there was only a small difference between N_mi_ and N_1macro_ (HR = 1.60 vs. HR = 1.54 in OS; HR = 2.03 vs. HR = 2.06 in CSS, respectively). It is suggested that N_mi_ was equally essential to assigning patients for more active treatment modalities. Moreover, recent studies have demonstrated that age at diagnosis and heterogeneous health backgrounds were significantly associated with the clinical decision-making for this population (8, 9).

Regarding race/ethnicity, it was recently determined to be associated with the prognosis of breast cancer (36–39). Especially, young black women with breast cancer had more adverse pathological factors and worse prognosis, when compared with white or Asian women. The potential intrinsic biological differences and socioeconomic status factors might be the contributors to these disparities. However, among elderly TNBC patients in our study, only Asian or Pacific Islander and American Indian/Alaska Native subgroups showed a survival advantage in OS and CSS, while there was no significant survival difference among black and white race patients. There were some possible explanations for our diverging findings. For instance, a study from San Miguel et al. suggested that insurance status played a pivotal role in breast cancer mortality, namely, uninsured women had the highest risk for breast cancer death, regardless of age (40). For this reason, insurance could be a pivotal factor but missed in our research which might influence the results we determined.

Based on the prognostic factors we determined, we further established an individualized predicting model for quantitatively analyzing the long-term OS and CSS probability for elderly primary operable TNBC patients. For example, one 75-year-old black TNBC (T_2_N_1_M_0_, moderate differentiation) patient after radiation without chemotherapy was met in the outpatient room. The physicians could calculate the 3-, 5-, and 7-year OS (78%, 66%, and 53%, respectively) and CSS (85%, 75%, and 73%, respectively) probability. The C-index derived from the training and validating cohorts supported that the two nomograms we developed had promising predicting value in clinical use. Moreover, the calibration curves and DCA graphically highlighted the accuracy and clinical utility of the model. The calculation outcome will help oncologists to choose adjuvant treatment regimens.

Reviewing recently published literature, our study partially confirmed their results and took it a step further (15, 16, 18, 26). To the best of our knowledge, this is the first population-based study to investigate the clinicopathological characteristics associated with the prognosis of elderly primary operable TNBC patients. The primary strength of our study is the large population-based sample size within 4,761 cases, which was significantly larger than previous studies on this topic (16, 33). Thus, the results, especially in terms of the favorable role in chemotherapy and radiotherapy for this population, provided further evidence-based suggestions for clinical practice guideline improvement. Moreover, the developed nomogram model included individuals of different races and ethnicities present in the US, which was different from other retrospective single-center designed studies.

Nevertheless, this observational study has some limitations which need to be mentioned. First, this is a retrospective study in which selection bias inevitably exists. Second, while ten pivotal variables were enrolled for analysis, some information regarding important confounders including but not limited to Ki-67 index (41) and 21-Gene Recurrence Score (21-GRS) (42) as well as medical comorbidities and functional status, which tend to correlate with age and the prognosis of breast cancer, is now unavailable from the SEER database. Third, the adjuvant chemotherapy regimens and cycles as well as the scope and dose of the radiotherapy were not given in the present study. Thus, whether chemotherapy and radiotherapy could benefit the elderly primary operable TNBC patients should be discussed cautiously and the determined results need to be interpreted carefully. Lastly, another limitation of this study is the lack of external independent cohorts which prohibits further enforcing the reliability and clinical application of the nomograms. Herein, a prospective, multicenter cohort study with more detailed indicators is urgently needed to further evaluate the independent prognostic factors we determined and get a higher level of evidence for clinical guideline updates.

## Conclusion

In conclusion, our results highlight that receiving adjuvant chemotherapy and radiotherapy could be favorable prognostic factors for elderly primary operable TNBC patients after local surgery. Besides, age, race, differentiation grade, T stage, and N stage were identified as the independent prognostic indicators for predicting the long-term survival of this population. The two novel nomograms could help physicians to evaluate the survival probability and make tailored clinical decisions in elderly TNBC patients. Nevertheless, these findings need to be further validated and explored in future studies.

## Data Availability Statement

The original contributions presented in the study are included in the article/**Supplementary Material**. Further inquiries can be directed to the corresponding authors.

## Author Contributions

All authors contributed to the conception and design of the study. ZT and YJ organized the database. YM, ZT, XZ, WX, and LZ performed the statistical analysis. All authors wrote the first draft of the manuscript. All authors wrote sections of the manuscript. All authors contributed to the manuscript revision and read and approved the submitted version.

## Conflict of Interest

The authors declare that the research was conducted in the absence of any commercial or financial relationships that could be construed as a potential conflict of interest.

## Publisher’s Note

All claims expressed in this article are solely those of the authors and do not necessarily represent those of their affiliated organizations, or those of the publisher, the editors and the reviewers. Any product that may be evaluated in this article, or claim that may be made by its manufacturer, is not guaranteed or endorsed by the publisher.
