# Supplementary material for: Prognostic Factors and Models for Elderly (≥70 Years Old) Primary Operable Triple-Negative Breast Cancer: Analysis From the National Cancer Database
Source: Front Endocrinol (Lausanne). 2022 Mar 17;13:856268. doi: 10.3389/fendo.2022.856268 (PMC8969604; doi:10.3389/fendo.2022.856268)
Supplement: Supplementary file 1 [file Table_1.docx]

**Table S1**. The specific value of clinicopathological factors in the nomogram (OS) in the training cohort.

| **Characteristics** | **Score** |
| --- | --- |
| **Age** |  |
| 70 | 0 |
| 75 | 17 |
| 80 | 33 |
| 85 | 50 |
| 90 | 67 |
| 95 | 83 |
| 100 | 100 |
| **Race** |  |
| White | 20 |
| Black | 27 |
| **^※^**Other | 0 |
| **Grade** |  |
| I | 0 |
| II | 45 |
| III/IV | 63 |
| **T stage** |  |
| T_mi+1a_ | 0 |
| T_1b_ | 16 |
| T_1c_ | 39 |
| T_2_ | 65 |
| T_3_ | 100 |
| **N stage** |  |
| N_0_ | 0 |
| N_1mi_ | 30 |
| N_1_ | 27 |
| **Radiation** |  |
| Not performed | 24 |
| performed | 0 |
| **Chemotherapy** |  |
| Not performed | 31 |
| performed | 0 |
| **Total point for 3- year OS** |  |
| 0.1 | 346 |
| 0.2 | 323 |
| 0.3 | 305 |
| 0.4 | 287 |
| 0.5 | 270 |
| 0.6 | 250 |
| 0.7 | 228 |
| 0.8 | 198 |
| 0.9 | 150 |
| **Total point for 5- year OS** |  |
| 0.1 | 314 |
| 0.2 | 291 |
| 0.3 | 273 |
| 0.4 | 256 |
| 0.5 | 238 |
| 0.6 | 219 |
| 0.7 | 196 |
| 0.8 | 166 |
| 0.9 | 119 |
| **Total point for 7- year OS** |  |
| 0.1 | 289 |
| 0.2 | 267 |
| 0.3 | 248 |
| 0.4 | 231 |
| 0.5 | 213 |
| 0.6 | 194 |
| 0.7 | 171 |
| 0.8 | 141 |
| 0.9 | 94 |

Notes: ^※^other: defined as the Asian/Pacific Islander and American Indian/Alaska Native;^＆^Grade: I: well differentiated, II: moderately differentiated, III/IV: poorly differentiated and undifferentiated.

Abbreviation: OS: overall survival.
