# Supplementary material for: Prognostic Factors and Models for Elderly (≥70 Years Old) Primary Operable Triple-Negative Breast Cancer: Analysis From the National Cancer Database
Source: Front Endocrinol (Lausanne). 2022 Mar 17;13:856268. doi: 10.3389/fendo.2022.856268 (PMC8969604; doi:10.3389/fendo.2022.856268)
Supplement: Supplementary file 2 [file Table_2.docx]

**Table S2**. The specific value of clinicopathological factors in the nomogram (CSS) in the training cohort.

| **Characteristics** | **Score** |
| --- | --- |
| **Age** |  |
| 70 | 0 |
| 75 | 7 |
| 80 | 15 |
| 85 | 22 |
| 90 | 29 |
| 95 | 37 |
| 100 | 44 |
| **Race** |  |
| White | 35 |
| Black | 38 |
| **^※^**Other | 0 |
| **Grade** |  |
| I | 0 |
| II | 28 |
| III/IV | 45 |
| **T stage** |  |
| T_mi+1a_ | 0 |
| T_1b_ | 15 |
| T_1c_ | 46 |
| T_2_ | 74 |
| T_3_ | 100 |
| **N stage** |  |
| N_0_ | 0 |
| N_1mi_ | 30 |
| N_1_ | 31 |
| **Radiation** |  |
| Not performed | 18 |
| performed | 0 |
| **Chemotherapy** |  |
| Not performed | 10 |
| performed | 0 |
| **Total point for 3- year OS** |  |
| 0.2 | 281 |
| 0.3 | 269 |
| 0.4 | 257 |
| 0.5 | 245 |
| 0.6 | 232 |
| 0.7 | 217 |
| 0.8 | 197 |
| 0.9 | 165 |
| **Total point for 5- year OS** |  |
| 0.1 | 281 |
| 0.2 | 266 |
| 0.3 | 254 |
| 0.4 | 242 |
| 0.5 | 230 |
| 0.6 | 217 |
| 0.7 | 202 |
| 0.8 | 182 |
| 0.9 | 149 |
| **Total point for 7- year OS** |  |
| 0.1 | 272 |
| 0.2 | 256 |
| 0.3 | 244 |
| 0.4 | 232 |
| 0.5 | 220 |
| 0.6 | 207 |
| 0.7 | 192 |
| 0.8 | 172 |
| 0.9 | 140 |

Notes: ^※^other: defined as the Asian/Pacific Islander and American Indian/Alaska Native;^＆^Grade: I: well differentiated, II: moderately differentiated, III/IV: poorly differentiated and undifferentiated.

Abbreviation: CSS: cancer-specific survival.
